# Structure-Guided Systems-Level Engineering of Oxidation-Prone Methionine Residues in Catalytic Domain of an Alkaline α-Amylase from *Alkalimonas amylolytica* for Significant Improvement of Both Oxidative Stability and Catalytic Efficiency

**DOI:** 10.1371/journal.pone.0057403

**Published:** 2013-03-15

**Authors:** Haiquan Yang, Long Liu, Hyun-dong Shin, Jianghua Li, Guocheng Du, Jian Chen

**Affiliations:** 1 The Key Laboratory of Carbohydrate Chemistry and Biotechnology, Ministry of Education, Jiangnan University, Wuxi, China; 2 Key Laboratory of Industrial Biotechnology, Ministry of Education, Jiangnan University, Wuxi, China; 3 School of Chemical and Biomolecular Engineering, Georgia Institute of Technology, Atlanta, Georgia, United States of America; 4 National Engineering Laboratory for Cereal Fermentation Technology, Jiangnan University, Wuxi, China; Universität Stuttgart, Germany

## Abstract

High oxidative stability and catalytic efficiency are required for the alkaline α-amylases to keep the enzymatic performance under the harsh conditions in detergent industries. In this work, we attempted to significantly improve both the oxidative stability and catalytic efficiency of an alkaline α-amylase from *Alkalimonas amylolytica* by engineering the five oxidation-prone methionine residues around the catalytic domain via a systematic approach. Specifically, based on the tertiary structure analysis, five methionines (Met 145, Met 214, Met 229, Met 247 and Met 317) were individually substituted with oxidation-resistant threonine, isoleucine and alaline, respectively. Among the created 15 mutants, 7 mutants M145A, M145I, M214A, M229A, M229T, M247T and M317I showed significantly enhanced oxidative stability or catalytic efficiency. In previous work, we found that the replacement of M247 with leucine could significantly improve the oxidative stability. Thus, these 8 positive mutants (M145A, M145I, M214A, M229A, M229T, M247T, M247L and M317I) were used to conduct the second round of combinational mutations. Among the constructed 85 mutants (25 two-point mutants, 36 three-point mutants, 16 four-point mutants and 8 five-point mutants), the mutant M145I-214A-229T-247T-317I showed a 5.4-fold increase in oxidative stability and a 3.0-fold increase in catalytic efficiency. Interestingly, the specific activity, alkaline stability and thermal stability of this mutant were also increased. The increase of salt bridge and hydrogen bonds around the catalytic domain contributed to the significantly improved catalytic efficiency and stability, as revealed by the three-dimensional structure model of wild-type alkaline α-amylase and its mutant M145I-214A-229T-247T-317I. With the significantly improved oxidative stability and catalytic efficiency, the mutant M145I-214A-229T-247T-317I has a great potential as a detergent additive, and this structure-guided systems engineering strategy may be useful for the protein engineering of the other microbial enzymes to fulfill industrial requirements.

## Introduction

The α-amylases (1, 4-α-D-glucan glucanohydrolase; EC 3.2.1.1) responsible for starch-hydrolyzing are being widely utilized in the food, textile and pharmaceutical industries [1]. Among the α-amylases, alkaline α-amylases are of particular importance due to the high catalytic capability under alkaline conditions, and find wide applications in detergent and textile industries [2], [3], [4].

Oxidative stability is one of the most important parameters for alkaline α-amylase, especially for its application in detergents and textile industries where the washing environment is oxidative [5]. However, the methionine and cysteine residues of α-amylase are easily oxidized to sulfoxide under the oxidative conditions [6]. The oxidation of methionines and cysteines increased the size of the side-chain and resulted in the steric obstruction in the active site, leading to the significant decrease even complete loss of the activity [7], [8]. In order to improve the oxidative stability of alkaline α-amylase, replacing oxidation-prone methionine residues with oxidation-resistant amino acids such as serine (Ser), leucine (Leu), isoleucine (Ile), threonine (Thr) and alanine (Ala) is a commonly used strategy. For example, the substitution of Met 208 in a *Bacillus* sp. TS-23 α-amylase with leucine enhanced the oxidative stability by 2.4-fold [8]. But in most cases the enhanced oxidative stability is at the cost of decreased catalytic efficiency or specific activity. For example, the specific activity of α-amylase from *Geobacillus stearothermophilus* US100 decreased from 1, 000 to 845 U/mg when Met 197 was substituted by alanine [7], and the *k*_cat_ of α-amylase from *Bacillus* sp. TS-23 was also decreased by 70% when simultaneously replacing Met 315 and Met 446 with leucine [9]. In our previous work, five methionines (Met 145, Met 214, Met 229, Met 247 and Met 317) of the alkaline α-amylase (AmyK) from alkaliphilic *Alkalimonas amylolytica* N10 were individually replaced with serine or leucine to enhance the oxidative stability [10], [11]. It was found that the replacement of Met 214 with serine can significantly improve the oxidative stability. However, the catalytic efficiency of the mutant M214S was decreased by 29% [10]. Therefore, significant improvement of both the oxidative stability and catalytic efficiency has remained as a challenge for engineering alkaline α-amylase. In this work, we attempted to significantly improve both the oxidative stability and catalytic efficiency of AmyK from *A. amylolytica* by systematically engineering five oxidation-prone methionine residues (Met 145, Met214, Met 229, Met 247 and Met 317) around the catalytic domain. This kind of protein engineering strategy used in this study may be useful for engineering the other microbial enzymes which have applications in industry.

## Materials and Methods

### Strains and plasmids

The AmyK gene (Accession No. AY268953) from alkaliphilic *A. amylolytica* N10 was synthesized based on the preferred codon usage of *Escherichia coli* by Sangon Biotech Co., Ltd (Shanghai, China). Plasmid pET-20b (+) was used as the expression vector of this synthetic gene, and *E. coli* JM109 was the host for cloning work, and *E. coli* BL21 (DE3) was the host for expression of AmyK. All the plasmids and strains were purchased from Sangon Biotech Co., Ltd (Shanghai, China).

### Site-directed mutagenesis

The recombinant plasmid was PCR amplified with mutagenic oligonucleotides (Table 1) using the MutanBEST Kit (TaKaRa, Dalian, China). Fragments amplified by PCR were purified and isolated on 0.8% (w/v) agarose gels after electrophoresis. The fragments were blunted by Blunting Kination Enzyme Mix (TaKaRa, Dalian, China) through the Blunting Kination reaction. The blunt-end fragments were ligated with Ligation Solution I (TaKaRa, Dalian, China). The reaction mixture was then transformed into competent cells of *E. coli* JM109. The transformants were selected on the Luria-Bertani (LB) agar plates containing 50 µg/mL ampicillin. The AmyK gene in the transformants was confirmed by PCR and checked by sequencing, and finally the recombinant plasmids were transformed into competent cells of *E. coli* BL21 (DE3) for expression.

**Table 1:** Oligonucleotide primers used for site-directed mutagenesis.

Mutant enzyme	Nucleotide sequence (5′→3′)^a^
M145T	CCATCGTATG(ACG)GGTGCGG
M214T	TATCTGATG(ACG)GGTGAAGACGTTGACT
M229T	AGGAGATG(ACG)AAGGCGTGGG
M247T	TTTCGTATG(ACG)GACGCGATTGC
M317T	CGGAGATATG(ACG)CGTTGGTGCGG
M145A	CCATCGTATG(GCG)GGTGCGG
M214A	TATCTGATG(GCG)GGTGAAGACGTTGACT
M229A	AGGAGATG(GCG)AAGGCGTGGG
M247A	TTTCGTATG(GCG)GACGCGATTGC
M317A	CGGAGATATG(GCG)CGTTGGTGCGG
M145I	CCATCGTATG(ATT)GGTGCGG
M214I	TATCTGATG(ATT)GGTGAAGACGTTGACT
M229I	AGGAGATG(ATT)AAGGCGTGGG
M247I	TTTCGTATG(ATT)GACGCGATTGC
M317I	CGGAGATATG(ATT)CGTTGGTGCGG

a : Nucleotides underlined correspond to the codons chosen for mutation. Nucleotides in parentheses replace the underlined nucleotides.

### Media and culture conditions

The seed culture of *E. coli* was done on a rotary shaker at 200 rpm and 37°C for 10 h in LB medium containing 5 g/L yeast extract, 10 g/L peptone and 5 g/L NaCl. The alkaline α-amylase production was performed in 250-mL shaker flasks containing 25 mL Terrific Broth (TB) medium containing 5 g/L glucose, 24 g/L yeast extract, 12 g/L peptone, 2.31 g/L KH_2_PO_4_, 16.43 g/L K_2_HPO_4_, and 0.1 g/L ammonium phosphate. The agitation speed and temperature were controlled at 200 rpm and 37°C, respectively. When the optical density at 600 nm (OD_600_) reached 0.6, the inducer isopropyl β-D-1-thiogalactopyranoside (IPTG) was added to a final concentration of 0.4 mM for induction of gene expression and then the cells were further cultivated at 25°C for 48 h.

### Purification of alkaline α-amylase

The culture broth was centrifugated at 8, 000×*g* for 15 min at 4°C and the obtained supernatant was used for enzyme purification. Solid ammonium sulfate was added to the supernatant to 70% of saturation at 0°C. The precipitate was collected and dissolved in glycine-NaOH buffer (20 mM, pH 9.0) and dialyzed overnight against the same buffer. After dialysis, the enzyme solution was filtered with microporous membrane (0.22 µm). The enzyme solution was then injected into an AKTA purifier (GE Healthcare, Houston, USA) through anion exchange (Q-Sepharose HP, Houston, USA). When the sample was injected, buffer A (20 mM phosphate buffer, pH 6.0) was used to adsorb the target enzyme to the column, and after eluting the unbound proteins, a linear elution was done by ramping buffer B (20 mM phosphate buffer (pH 6.0) and 1 M NaCl) from 0 to 100%. The flow rate of the buffer was 1.0 mL/min. The fractions were collected for activity assay and sodium dodecyl sulfate-polyacrylamide gel electrophoresis (SDS-PAGE) analysis.

### Enzyme assays

AmyK activity was determined by measuring the amount of reducing sugar released during hydrolysis of 1% (w/v) soluble starch in glycine-NaOH buffer (20 mM, pH 9.5) at 50°C for 5 min. A control without enzyme addition was used. The amount of reducing sugar was measured with a modified dinitrosalicylic acid method [12]. One unit of enzyme activity was defined as the amount of enzyme that released 1 µmol of reducing sugar as glucose per min under the assay condition. The protein concentration was measured by the Bradford method [13] with bovine albumin (Sangon Biotech, Shanghai, China) as the standard.

### Construction of three-dimensional (3-D) structure model

Homology modeling was conducted with the Swiss Model server (http://swissmodel.expasy.org/) to predict the structures of the AmyK and its mutants [14]–[17]. In this work, the sequence of AmyK showed low similarity to the other known α-amylases, and had the highest identity of 55.9% with the α-amylase AmyB (3bc9) from *Halothermothrix orenii* [16], which was used as the template for homology modeling. In addition, it was found that the starch binding domain of AmyK had no identity with that of template α-amylase AmyB (3bc9). Therefore, the amino acid sequences used to construct 3-D structure model were catalytic domain (Domain A, Domain B and Domain C) and did not include the sequence of starch binding domain. For the mutants, the mutation amino acid residues of sequence were first substituted with the corresponding amino acids, and then the 3-D structure models of mutants were constructed by the Swiss Model server (http://swissmodel.expasy.org/) with the α-amylase AmyB (3bc9) from *H. orenii* as a template. The sequence alignment of the AmyK and the template was done by Vector NTI Explorer (Invitrogen, Frederick, USA), and the detailed sequence alignment was attached in the Supplementary Materials (Fig. S1). The estimated absolute model quality (QMEAN Z-Score) of modeling structure was −2.47. The template and modeled structure was overlapped by the Discovery Studio 2.5 (Accelrys, San Diego, USA). The root-mean-square deviation (RMSD) of the modeling structure was calculated with Swiss-PdbViewer 4.0.4 (SIB Swiss Institute of Bioinformatics, Geneva, Swiss), and the RSMD of alpha carbon atoms for the modeling structure was 33.76 Å. The number of hydrogen bonds and salt bridges in the enzyme was calculated by Discovery Studio 2.5 (Accelrys, San Diego, USA).

### Influence of mutation on oxidative stability

The mutants were incubated with 500 mM H_2_O_2_ in glycine-NaOH buffer (20 mM, pH 9.0) at 35°C for 1 h. After 1 h incubation in H_2_O_2_, catalase (10, 000 U/mL) was immediately added to the samples to a final concentration of 2, 000 U/mL to quench the remained H_2_O_2_. The starting concentration for the alkaline α-amylase was 1.51 µmol (protein)/mol (H_2_O_2_) when incubation with H_2_O_2_. Samples (200 µL) were then withdrawn to measure the residual activity under the standard assay conditions.

### Influence of mutation on kinetic parameters

The kinetic parameters (*K*_m_, *V*_max_, *k*_cat_ and *k*_cat_/*K*_m_) of the AmyK and its mutants were determined in glycine-NaOH buffer (20 mM, pH 9.5) at 50°C. Assays were performed with active enzyme at a fixed starting level of 16 U/mL and soluble starch at concentrations ranging from 2.9 to 29×10^−3^ mol/L. Enzyme kinetic parameters *K*_m_ and *V*_max_ were estimated from Eadie-Hofstee plots.

### Effect of pH and temperature on activity and stability

To estimate the optimal pH of mutant alkaline α-amylases, the purified proteins were incubated in sodium phosphate buffer (20 mM, pH 7.0–8.0), Tris-HCl buffer (20 mM, pH 8.0–9.5), glycine-NaOH buffer (20 mM, pH 9.5–11.0), and Ca(OH)_2_ buffer (20 mM, pH 12.0). The pH stability of alkaline α-amylase was determined at pH ranging from 8.0 to 11.0 at 25°C for 24 h. After incubation, the alkaline α-amylase activity was measured at pH 9.5 and 50°C.

The activities of mutants were determined at 35, 40, 45, 50, 55, 60 and 65°C, respectively, to determine the optimal temperature. The thermal stability of alkaline α-amylase was determined at 60°C in glycine-NaOH buffer (20 mM, pH 9.0). The activation energy (*E*_a_) was determined in the temperature range from 20 to 50°C with the Arrhenius equation ln (*k*) = (−*E*_a_/*RT*)+ln (*A*). The reaction rate constant (*k*) was calculated from the equation ln (*c*) = −*kt*+ln (*c*_0_), where *c* is the concentration of the substrate (soluble starch), and *c*_0_ is the initial concentration of the substrate (10 g/L, w/v).

### Differential scanning calorimeter (DSC), Circular dichroism (CD), fluorescence spectroscopy analysis

The melting temperatures of the AmyK and its mutants were detected on Q2000 DSC (TA Instruments-Waters LLC, New Castle, USA) at 50 mg/mL protein concentration in 20 mM Tris-HCl buffer (pH 9.0). The temperature was increased at a rate of 10°C/min over the range of 20 to 90°C. The CD spectra was measured on MOS-450/AF-CD-STP-A (Bio-Logic, Grenoble, France) at a protein concentration of 0.1 mg/mL in a 1 cm path-length quartz cuvette in 20 mM Tris-HCl buffer (pH 9.0). In order to minimize signal baseline drift, the spectropolarimeter and xenon lamp were warmed up for at least 30 min prior to each experiment. Ellipticity data of the enzymes in the band of 200–250 nm were collected, and the spectrum of a buffer blank was subtracted. The lengths of α-helix and β-sheet were determined online by the DichroWeb (http://dichroweb.cryst.bbk.ac.uk/html/process.shtml). The fluorescence emission was detected on SP-2558 (Roper Scientific, Trenton, USA) at 1 mg/mL protein concentration in 20 mM Tris-HCl buffer (pH 9.0). The range was from 300 to 420 nm and the excitation wavelength used to record fluorescence emission spectra was 280 nm.

### Effect of surfactants and detergents on activity and stability

The effects of surfactants Tween 20, Tween 60, Tween 80, Triton X-100, and sodium dodecyl sulfate (SDS) on enzyme stability were studied by incubating enzyme at 40°C for 1 h. The concentration of surfactants and alkaline α-amylase were 10% (w/v) and 16 U/mL, respectively. The activity of enzyme without surfactant addition was taken as 100%.

Compatibility of enzyme with solid detergents was studied with laundry solid soap (Nice, China), toilet soap (Safeguard, USA), washing powder-1 (Tide, USA), and washing powder-2 (Nice, China). Compatibility of enzyme with liquid detergents was studied with laundry detergent-1 (Blue noon, China), laundry detergent-2 (Liby, China), liquid detergent-1 (Nice, China), and liquid detergent-2 (Liby, China). The detergents were first diluted in tap water to give a concentration of 70 mg/mL for solid detergents and 10% (v/v) for liquid detergents. The proteases from the detergents were inactivated by incubating the detergents at 65°C for 1 h before addition. When enzyme activity was determined, the detergents were diluted to give a final concentration of 7 mg/mL for solid detergents and 1% (v/v) for liquid detergents to simulate washing conditions [7]. The enzyme activity of the control without detergent addition was taken as 100%.

## Results and Discussion

**Figure 1:**
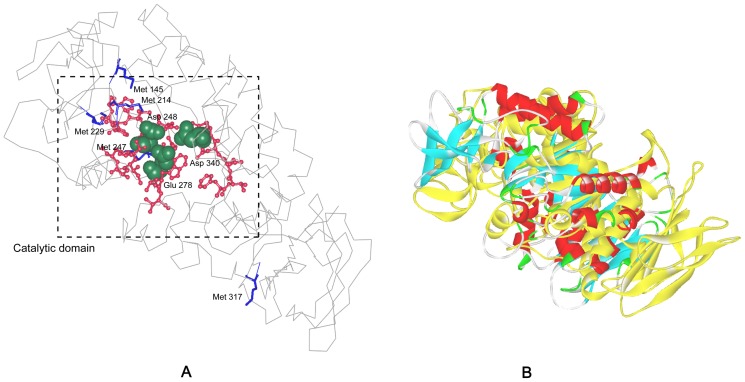
The structure model of alkaline α-amylase from *A. amylolytica* and the structure of both template and modeled structure overlapping. A: The model structure of alkaline α-amylase was constructed with the crystal structure of α-amylase AmyB (3bc9) as a template. The catalytic residues Asp248, Glu278, and Asp340 are shown in “CPK (Corey-Pauling-Koltum)” representation. The blue “sticks” indicated the mutant positions. The red “ball and stick” indicated the conserved regions. B: The overlapping of template and modeled α-amylase structure. The yellow is the template (AmyB) structure, and the other is the modeled structure of alkaline α-amylase.

As revealed by the 3-D structure model of AmyK, Met 145, Met 214, Met 229 and Met 247 were located at the cavity of catalytic domain (Fig. 1). Moreover, although Met 317 was not in the cavity of active domain, it was close to the active site and exposed to the surface, and its oxidation also can decrease the oxidation stability of *A. amylolytica* alkaline α-amylase. We have shown that the oxidative stability can be enhanced to a certain extent by replacing these five methionine residues with serine or leucine, while the catalytic efficiency of nearly all mutants decreased [10], [11].

In this work, we adopted a systems protein engineering strategy, namely, combining the first round of one-point mutations and the second round of combinational mutations, to achieve the significant improvement of both oxidative stability and catalytic efficiency. In the first round of one-point mutations, the five methionine residues were individually substituted with oxidation-resistant amino acids isoleucine, threonine and alanine, yielding fifteen mutants.

### Influence of the first round one-point mutation on oxidative stability and catalytic efficiency

**Figure 2:**
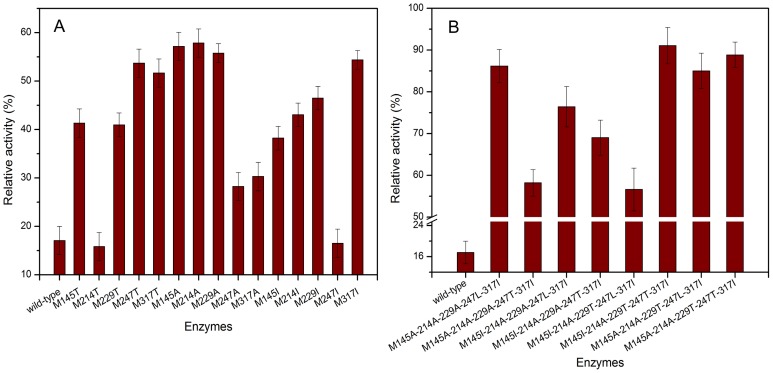
Oxidative stability of wild-type and mutant proteins. The relative activity (%) was determined and compared with the activity without the addition of H_2_O_2_. A: Oxidative stability of the one-site mutant enzymes. B: Oxidative stability of eight five-site mutant enzymes.

When recombinant strain was cultured in shaker flask, the yield of the wild-type was the same with that of the mutants (20 U/mL). Fig. 2A shows the oxidative stability of fifteen mutants created in the first round of one-point mutations. The AmyK was susceptible to oxidation and retained 17% of the original activity when incubation with 500 mM H_2_O_2_ at 35°C for 1 h. Under the same conditions, the mutants M145T, M229T, M247T, M317T, M145A, M214A, M229A, M145I, M214I, M229I and M317I exhibited enhanced resistance to oxidation, and in particular, the mutants M145A, M214A, M229A, M247T and M317I retained 57, 58, 56, 54 and 54% of original activity, respectively.

These results confirmed the importance of methionine residues in oxidative sensitivity of amylase. The oxidation of methionine can decrease the activity and the substitution of methionine residue by an anti-oxidative amino acid may enhance the oxidative stability of the enzyme. For example, Met 197, which was situated close to the active site of amylase from *G. stearothermophilus*, has been shown to be involved in oxidative inactivation, and the introduction of a non-sulfur-containing amino acid at this position greatly reduced the oxidative sensitivity of the enzyme [7]. In this work, as suggested by the constructed model, Met 145, Met 214, Met 229, and Met 247 were situated in the cavity of active site. The oxidative stability of the mutants M145A, M214A, M229A and M247T was increased significantly. The surface-exposed methionine residues in proteins were also susceptible to oxidation and here it was shown that the oxidative stability of the enzyme was also enhanced when Met 317 was replaced by Thr or Ile.

The influence of one-point mutation on specific activities of enzyme was determined. The specific activity of the mutants M229T and M247T was increased from 1.60×10^3^ U/mg of the wild-type enzyme to 1.79× and 1.98×10^3^ U/mg, respectively. Table 2 shows the influence of one-point mutations on the catalytic efficiency. The catalytic constants (*k*_cat_) of the mutants M229T and M247T were increased from 8.3×10^2^ s^−1^ of wild-type enzyme to 10.1× and 11.7×10^2^ s^−1^, respectively. The *k*_cat_/*K*_m_ of the mutants M145I and M229T was improved from 3.2×10^4^ L mol^−1^ s^−1^ of the AmyK to 4.5× and 6.2×10^4^ L mol^−1^ s^−1^, respectively. The *k*_cat_/*K*_m_ of the mutant M247T was not changed compared with that of AmyK. However, the catalytic efficiency of the other one-point mutants was decreased compared with that of AmyK. The *K*_m_ value indicated the substrate binding capacity of the enzyme, and the specific activity indicated the activity per protein concentration of enzyme. The substrate binding capacity was independent with the specific activity of enzyme. The mutation might cause a decrease in both *K*_m_ value and specific activity, or a decrease in one and increase in the other. For example, Lin et al. found that when eight Met residues of the truncated *Bacillus* sp. TS-23 α-amylase was replaced by Leu, the *K*_m_ value of all mutants decreased, and the specific activity of the mutants (M17L, M104L, M208L, M214L, M229L and M324L) increased, but that of the mutants M315L and M446L decreased [9].

**Table 2:** Kinetic parameters of wild-type and one-point mutants.

Enzyme	*K*_m_ (mM)	*k*_cat_ (·10^2^ s^−1^)	*k*_cat_/*K*_m_ (·10^4^ L mol^−1^ s^−1^)
Wild-type	26.4±2.3	8.3±0.3	3.2±0.2
M145T	10.5±2.0	1.9±0.2	1.8±0.2
M214T	15.4±2.8	2.8±0.2	1.8±0.2
M229T	16.3±1.5	10.1±0.4	6.2±0.3
M247T	30.4±2.8	11.7±0.6	3.8±0.2
M317T	27.8±3.1	8.3±0.4	3.0±0.2
M145A	27.3±3.0	7.3±0.3	2.7±0.2
M214A	28.8±2.4	8.2±0.2	2.8±0.2
M229A	21.5±4.1	6.8±0.5	3.2±0.3
M247A	10.8±2.3	1.6±0.1	1.5±0.2
M317A	26.4±2.4	7.7±0.2	2.8±0.2
M145I	18.5±1.3	8.4±0.2	4.5±0.2
M214I	25.4±2.8	7.9±0.4	3.0±0.2
M229I	10.8±2.4	1.6±0.2	1.5±0.2
M247I	7.4±1.6	1.5±0.2	2.0±0.2
M317I	26.1±2.4	7.8±0.3	3.0±0.2
M247L*	13.7±1.1	9.4±0.3	6.8±0.3

* The data was referenced from our previous work [11].

It was indicated that the above single mutations were important for improvement of catalytic efficiency of enzyme. The increased substrate binding ability (as indicated by the decreased *K*_m_) of the mutants contributed to the increase of the *k*_cat_/*K*_m_, while the increase in the substrate affinity may result from changes in electrostatic interactions around the catalytic residues after mutation [11]. In most cases, site-directed mutagenesis has a negative influence on catalytic efficiency. For example, the specific activities of the mutants M145L, M214L, M229L, and M317L decreased, while that of the mutant M247L increased compared with that of the AmyK [11].

By comprehensive analysis of one-point mutations on the oxidative stability and catalytic efficiency, it could be found that the mutants M145A, M145I, M214A, M229A, M229T, M247T and M317I had much higher oxidative stability or catalytic efficiency than the wild-type enzyme. In our previous work, it was found that the substitution of M247 with leucine could significantly improve the oxidative stability [11]. Therefore, there are total eight positive one-point mutants, namely, M145A, M145I, M214A, M229A, M229T, M247T, M247L and M317I for the second round of combinational mutations.

### Effect of the second round of combinational mutations on oxidative stability and catalytic efficiency

In order to significantly enhance both oxidative stability and catalytic efficiency, the combinational mutations were conducted by systematically combining the eight positive mutations mentioned above, and 85 combinational mutants (25 two-point mutants, 36 three-point mutants, 16 four-point mutants and 8 five-point mutants, as shown in Table S1) were obtained. It was found that the five-point mutants showed higher oxidative stability and catalytic efficiency than the other combinational mutants (data not shown), and thus here we emphatically discussed the biochemical properties of these 8 five-site mutants (M145A-214A-229A-247L-317I, M145A-214A-229A-247T-317I, M145I-214A-229A-247L-317I, M145I-214A-229A-247T-317I, M145I-214A-229T-247L-317I, M145I-214A-229T-247T-317I, M145A-214A-229T-247L-317I and M145A-214A-229T-247T-317I). The purity of the wild-type and these 8 mutants was confirmed by the SDS-PAGE, and the proteins after mutation was about 60 kDa (Fig. S2). Fig. 2B shows the oxidative stability of the 8 five-site mutants, respectively. It could be seen that the oxidative stabilities of five-point mutants were significantly enhanced, and in particular, the mutant M145I-214A-229T-247T-317I retained 91.3% of original activity during the incubation with 500 mM H_2_O_2_ in glycine-NaOH buffer (20 mM, pH 9.0) for 1 h, and the residual activity was enhanced by 5.4-fold compared with that of the wild-type enzyme. When the Met 197 of AmyUS100ΔIG (α-amylase from *G. stearothermophilus* US100) was substituted by Ala, 70% of mutant AmyUS100ΔIG/M197A activity was retained after 1 h of treatment with 1.8 M H_2_O_2_ at pH 5.6 in 50 mM acetate buffer [7]. It was indicated that the different methionine residues substituted by different anti-oxidant amino acids had different influence on the anti-oxidation of alkaline α-amylase in this work. Moreover, due to the synergetic interaction of the five sites, after combinational mutation, the oxidative stability was more significantly enhanced compared with that of single mutants. For example, the four-point mutant M145/214/247/317L retained 91.2% of its original activity [11].

Table 3 shows the catalytic efficiency of the 8 five-point mutants, respectively. The *K*_m_ values of the mutants M145I-214A-229A-247T-317I, M145I-214A-229T-247T-317I, M145A-214A-229T-247L-317I and M145A-214A-229T-247T-317I were decreased from 26.4 mM of wild-type enzyme to 4.4, 16.9, 20.0 and 23.3 mM, respectively, indicating that the mutants had improved substrate affinities than the wild-type enzyme. The *k*_cat_ of the mutants M145A-214A-229A-247L-317I, M145I-214A-229T-247L-317I, M145I-214A-229T-247T-317I, M145A-214A-229T-247L-317I and M145A-214A-229T-247T-317I was increased from 8.3×10^2^ s^−1^ of the wild-type to 9.0, 9.3, 16.3, 9.5 and 9.8×10^2^ s^−1^, respectively. The catalytic efficiency (*k*_cat_/*K*_m_) of the mutants M145A-214A-229A-247L-317I, M145I-214A-229A-247T-317I, M145I-214A-229T-247T-317I, M145A-214A-229T-247L-317I and M145A-214A-229T-247T-317I was consequently improved, especially the mutant M145I-214A-229T-247T-317I showed an increase of *k*_cat_/*K*_m_ from 3.2×10^4^ L mol^−1^ s^−1^ of the wild-type enzyme to 9.6×10^4^ L mol^−1^ s^−1^. In our previous work, it was found that the *k*_cat_/*K*_m_ of the mutant M145/214/247/317L with high oxidative stability was not improved compared with that of wild-type enzyme [11]. The specific activity of the mutant M145I-214A-229T-247T-317I was also increased from 1.60×10^3^ U/mg of wild-type enzyme to 3.93×10^3^ U/mg. After the Met 208 in a truncated *Bacillus* sp. TS-23 α-amylase was replaced by Leu, the specific activity of mutant increased from 159.8 U/mg of wild-type enzyme to 219.2 U/mg [9]. But after mutation, the specific activity of AmyUS100ΔIG/M197A mutant decreased from 1,000 U/mg of AmyUS100ΔIG to 845 U/mg [7]. For combinational mutants M145A-214A-229A-247L-317I and M145A-214A-229A-247T-317I, three methionine residues were replaced by Ala, and the *K*_m_ values of these two mutants significantly decreased. This indicated that the substitutions with smaller amino acid residues such as Ser and Ala caused a drastic decrease in the substrate binding capability, while substitutions with the bulky Thr and Ile had little influence [7]. The catalytic efficiency of the mutant M145I-214A-229T-247T-317I was greatly enhanced compared to that before mutation.

**Table 3:** Kinetic parameters of wild-type and eight five-point mutant enzymes.

Enzyme	*K*_m_ (mM)	*k*_cat_ (·10^2^ s^−1^)	*k*_cat_/*K*_m_ (·10^4^ L mol^−1^ s^−1^)
Wild-type	26.4±2.3	8.3±0.3	3.2±0.2
M145A-214A-229A-247L-317I	38.4±2.5	9.0±0.2	2.3±0.2
M145A-214A-229A-247T-317I	45.3±2.4	8.5±0.3	1.9±0.2
M145I-214A-229A-247L-317I	32.9±1.5	3.4±0.1	1.0±0.2
M145I-214A-229A-247T-317I	4.4±1.2	1.6±0.1	3.6±0.2
M145I-214A-229T-247L-317I	27.5±1.8	9.3±0.6	3.4±0.2
M145I-214A-229T-247T-317I	16.9±1.0	16.3±0.4	9.6±0.3
M145A-214A-229T-247L-317I	20.0±1.6	9.5±0.4	4.8±0.2
M145A-214A-229T-247T-317I	23.3±1.8	9.8±0.3	4.2±0.2

**Figure 3:**
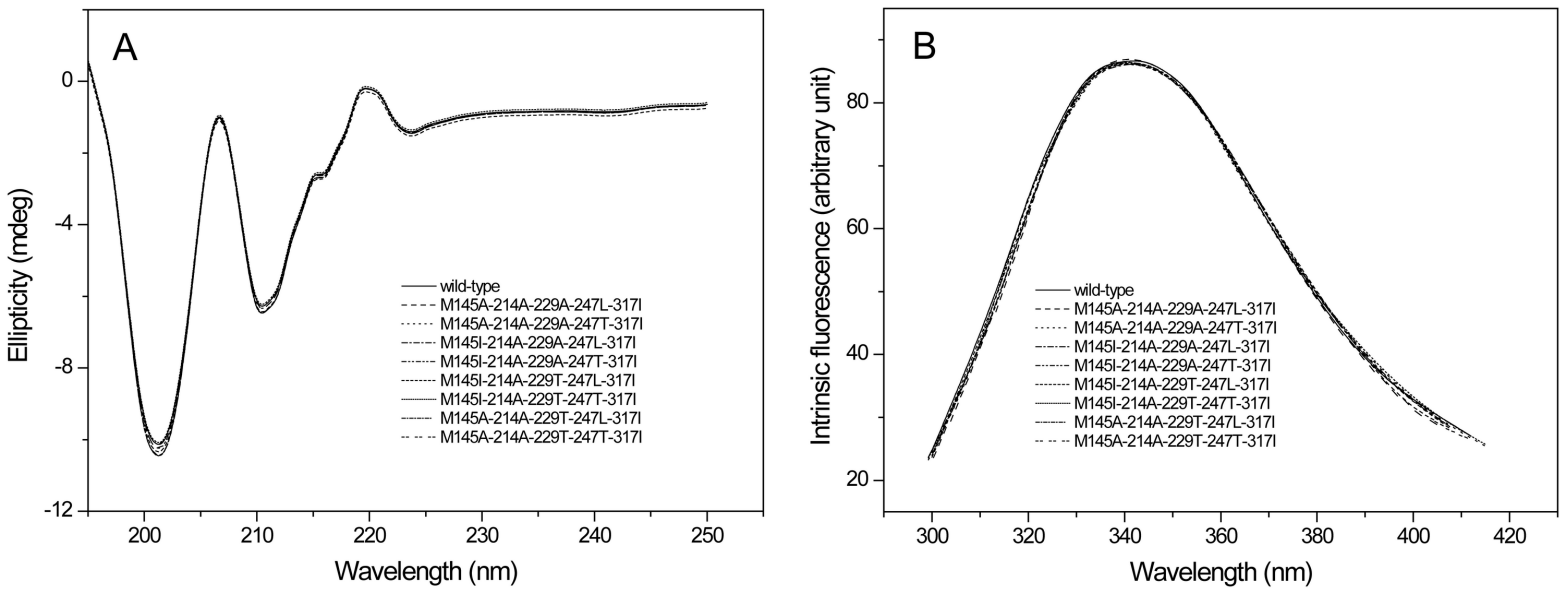
The circular dichroism (CD) and fluorescence spectroscopy of the wild-type and mutant proteins. A: CD spectral; B: The fluorescence spectroscopy.

**Figure 4:**
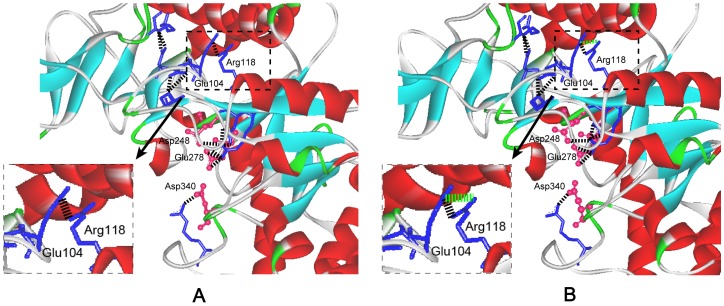
The change of the salt bridge around active sites of mutant M145I-214A-229T-247T-317I. The catalytic residues Asp^248^, Glu^278^ and Asp^340^ are shown in red “ball and stick” representation. The blue “sticks”: residues forming salt bridge around the active site. The α helices and β sheets are shown in red and cyan, respectively. A: The salt bridge around active sites of wild-type enzyme. The black “dotted line” is the salt bridge. B: The change of the salt bridge around active sites of the mutant. The newly formed salt-bridge between Arg 118 and Glu 104 is included in the “dotted frame”. The green “dotted line” is the new salt bridge formed after mutation.

The CD spectral and fluorescence spectroscopy were further conducted to evaluate the influence of mutations on the structure of enzymes. As shown in Fig. 3, all the eight five-point mutants had the same CD spectral and fluorescence spectroscopy with those of wild-type enzyme, indicating that the mutations had little influence on the secondary and tertiary structure of enzyme. Salt bridge has important effects on catalytic efficiency of enzymes [18], [19]. The thermostable NADPH:FMN oxidoreductase from *B. subtilis* was stabilized by four salt bridges formed by the side chains of residues Lys 109 and Asp 137 [18]. The Glu 121-Lys 319 salt bridge between catalytic and N-terminal domains is important for the catalytic efficiency and stability of *E. coli* aminopeptidase N [19]. In this work, based on the 3-D structure model of the wild-type and the mutant M145I-214A-229T-247T-317I (Fig. 4), it was found that a new salt bridge formed between Arg 118 and Glu 104 around the active site, and this may be one reason for the significantly improved catalytic efficiency of M145I-214A-229T-247T-317I.

### Influence of combinational mutations on the pH stability

**Figure 5:**
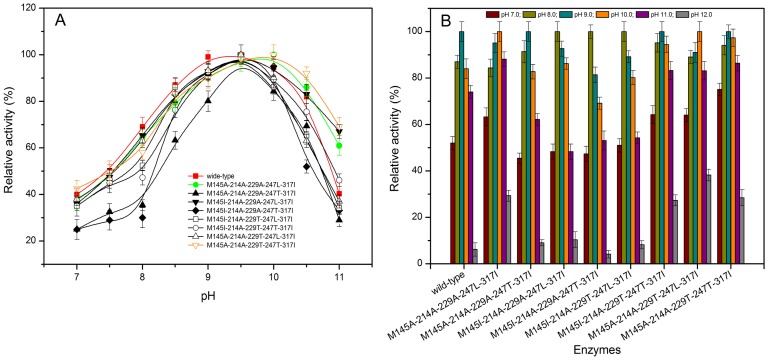
Effect of pH on activity and stability of eight five-site mutant enzymes. A: Effect of pH on activity of mutants. B: Effect of pH on stability of mutants.

As shown in Fig. 5A, the optimal pHs of the mutants M145A-214A-229A-247L-317I and M145A-214A-229T-247T-317I were shifted from 9.5 of the wild-type to 10.0. The stable pH range of the mutants M145A-214A-229A-247L-317I, M145I-214A-229T-247T-317I, M145A-214A-229T-247L-317I and M145A-214A-229T-247T-317I were also shifted from 8.0-10.0 of the wild-type to 8.0-11.0 (Fig. 5B). The stable pH ranges of the other mutants (M145A-214A-229A-247T-317I, M145I-214A-229A-247L-317I, M145I-214A-229A-247T-317I and M145I-214A-229T-247L-317I) were not changed as compared to that of the wild-type.

**Figure 6:**
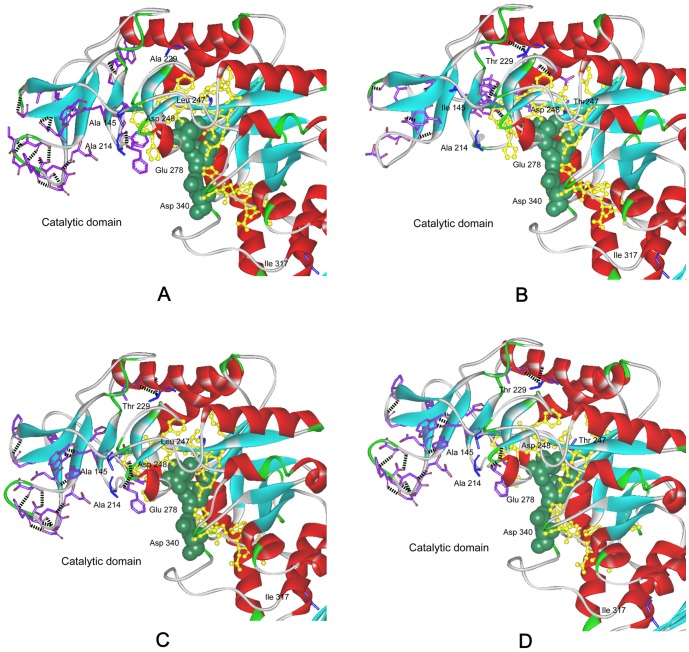
Local hydrogen bonding network of the five-site mutants. The active site is shown in a green CPK (Corey-Pauling-Koltun) representation. The yellow “ball and stick” indicate the conserved regions. The blue “stick” indicates the mutation site. The purple “stick” is the residue that forms new hydrogen bonding after mutation. The black “dotted line” is the hydrogen bonding. The α helices and β sheets are shown in red and cyan, respectively. A: M145A-214A-229A-247L-317I; B: M145I-214A-229T-247T-317I; C: M145A-214A-229T-247L-317I; D: M145A-214A-229T-247T-317I.

Hydrogen bonding network plays an important role for the pH stability of amylase, and a strengthened hydrogen bonding interaction can increase pH stability [20]. For example, after the replacement of Leu 134 by Arg, the stability of the *B. licheniformis* α-amylase was enhanced due to a newly formed hydrogen bonding network [20]. In this work, the influence of combinational mutations on the number and locations of hydrogen bonds around the conserved regions of the enzyme was examined. As shown in Fig. 6, the numbers of total hydrogen bonds of the mutants M145A-214A-229A-247L-317I, M145I-214A-229T-247T-317I, M145A-214A-229T-247L-317I and M145A-214A-229T-247T-317I were increased from 437 of the wild-type to 452, 449, 450 and 450, respectively. The increase of the hydrogen bonds may contribute to the pH stability of these mutants.

### Influence of combinational mutations on the thermal stability

**Figure 7:**
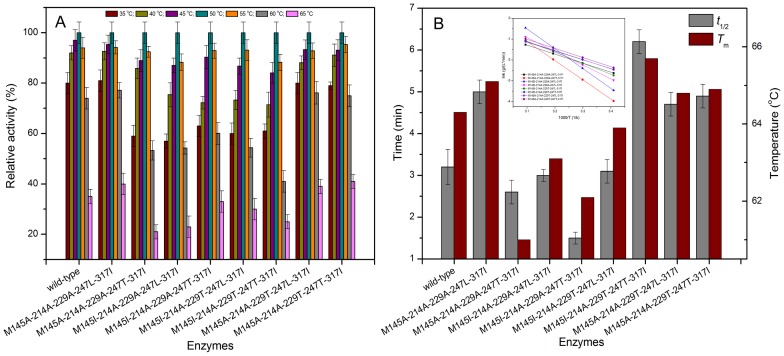
Effect of temperature on the activity and stability of eight five-site mutant enzymes. A: Effect of temperature on the activity of mutant enzymes. B: Effect of temperature on the stability of mutant enzymes. The inset presents the Arrhenius plot of the logarithm of the *k* values against the reciprocal of the absolute temperature (*T*). The values shown are activation energies calculated from the plot.

Fig. 7A shows the effects of temperature on the activity of 8 five-site mutants. The optimal temperature for all mutants was 50°C, the same to that of the wild-type. The activation energies of the mutations (M145A-214A-229A-247L-317I, M145I-214A-229T-247T-317I and M145A-214A-229T-247L-317I) were decreased, while those of the other mutants were increased compared with that (36.1 KJ/mol) of wild-type (Fig. 7B). The half-life times (*t*_1/2_) at 60°C of the mutants M145A-214A-229A-247L-317I, M145I-214A-229T-247T-317I, M145A-214A-229T-247L-317I and M145A-214A-229T-247T-317I were about 1.6-, 1.9-, 1.5- and 1.5-fold of that of the wild-type (Fig. 5B). Meanwhile, the melting temperature (*T*_m_) of the mutants M145A-214A-229A-247L-317I, M145I-214A-229T-247T-317I, M145A-214A-229T-247L-317I and M145A-214A-229T-247T-317I were improved by 0.8, 1.4, 0.5 and 0.6°C compared to that of the wild-type.

The thermostability of the mutants (M145A-214A-229A-247L-317I, M145I-214A-229T-247T-317I, M145A-214A-229T-247L-317I, and M145A-214A-229T-247T-317I) was enhanced after combinational mutations, especially the mutant M145I-214A-229T-247T-317I. The hydrogen bonds would appear to be responsible for maintaining the enzyme stability at high temperature [21]. The increased hydrogen bonding network may improve the thermal stability of proteins [22]. At high temperature, the enzyme seems unable to retain the tightly coiled, thermostable, and catalytically active structure in the absence of proper hydrogen bonds [6]. As shown in Fig. 6, the five-point mutants M145A-214A-229A-247L-317I, M145I-214A-229T-247T-317I, M145A-214A-229T-247L-317I, and M145A-214A-229T-247T-317I showed the increased number of hydrogen bonds, and this might make the mutant proteins more stable at high temperature. Moreover, the salt bridge also played a critical role for the stability of enzyme [6], [17], [22]. The salt bridge around the active site of M145I-214A-229T-247T-317I mutant was increased from 20 of the wild-type to 21 (Fig. 4), and this may further contribute to the thermostability of the mutant M145I-214A-229T-247T-317I.

### Effect of surfactants and detergents on the stability and activity of combinational mutants

**Figure 8:**
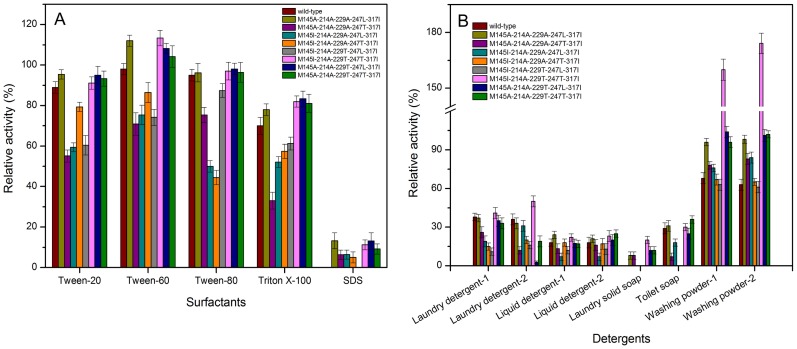
Effects of surfactants and detergents on the eight five-site mutant enzymes. A: Effects of surfactants on stability of mutants. B: Effects of detergents on activity of the mutants.

To evaluate the mutants' potential utility in the detergent industries, the activity of enzymes was evaluated in the presence of surfactants, including Tween 20, Tween 60, Tween 80, Triton X-100 and SDS. As shown in Fig. 8A, compared to that of wild-type, the activities of the mutants (M145A-214A-229A-247L-317I, M145I-214A-229T-247T-317I, M145A-214A-229T-247L-317I and M145A-214A-229T-247T-317I) were increased after incubation at 35°C for 1 h in the presence of the above surfactants (10%), while those of the other mutants were decreased. As an anionic surfactant, SDS may have altered electrostatic interactions with the enzyme, causing a change in the enzyme activity [23]. For nonionic surfactants such as Tween 20, Tween 60, Tween 80 and Triton X-100, without electrostatic interactions with the enzyme, the effect on the enzyme activity might be due to local structural rearrangements of the critical residues in the active site as a consequence of the mutations [23], [24]. Compared with the water, the solvents (e.g. ethylene glycol and trifuoroethanol) were found to lead to more stabilization of proteins structures due to the strengthened hydrogen bonding network [25]. In this work, as mentioned above, the number of hydrogen bonds of mutants increased compared to that of the wild-type, and it was hypothesized that this might be the main reason for the enhanced stability of mutants in the nonionic surfactants.

The activities of the enzymes in the presence of different solid and liquid detergents were also determined. As shown in Fig. 8B, the wild-type and mutant enzymes were found to be not stable in the presence of liquid detergents, and the enzyme activities decreased to less than 50% in the presence of laundry detergents or liquid detergents. However, in the presence of solid washing powder, the activities of the mutants M145A-214A-229A-247L-317I, M145I-214A-229T-247T-317I, M145A-214A-229T-247L-317I and M145A-214A-229T-247T-317I were improved compared to that of wild-type, especially the mutant M145I-214A-229T-247T-317I, the activity of which was improved by 2.4- and 2.8-fold in the presence of washing powder-1 and washing powder-2, respectively.

The laundry detergent was non-anionic detergent and others were anionic detergents. When the non-anionic laundry detergent was added, the activity of enzymes decreased, and this was similar with the addition of anionic liquid detergent. It might be hypothesized that the non-anion or anion was not the main reason that affected the activity of enzymes. Since the optimum pH of alkaline α-amylases in this work was in alkaline range, the activity of enzymes greatly decreased in the presence of liquid detergents having acidic pH (5.5–8.0). Due to the solid detergents had the pH ranging from 9.0 to 11.0, the enzymes were stable when the solid detergents were added. These might be the main reason why the enzymes were more compatible with solid washing powder than liquid detergents. This observation agreed well with other studies where the activity of AmyUS100ΔIG/M197A from *Geobacillus stearothermophilus* was increased by 10∼20% after incubation in the presence of Lav^+^ and Nadhif (detergents) [7].

## Conclusions

In this work, we significantly improved both the oxidative stability and catalytic efficiency by a systems protein engineering strategy based on the protein structure information. Among the constructed mutants, the mutant M145I-214A-229T-247T-317I showed a 5.4-fold increase in oxidative stability and a 3.0-fold increase in catalytic efficiency. In addition, the specific activity, alkaline stability and thermal stability of this mutant were also improved by different extents simultaneously. As revealed by the 3-D structure model, the increased salt bridge and hydrogen bonds around the catalytic domain may contribute to the improved catalytic efficiency and stabilities of the mutant M145I-214A-229T-247T-317I, which has a great potential in the detergents industries, especially as ingredients of washing powder. Furthermore, the systems protein engineering strategy developed in this work also may be useful for the engineering of the other industrial enzymes to improve their performances and fulfill industrial requirements. In future work, the other oxidation prone amino acids in the *A. amylolytica* alkaline α-amylase will be systematically engineered to improve catalytic performance and stabilities to meet requirements of industrial applications.

## Supporting Information

Figure S1 **The SDS-PAGE analysis of the purified wild-type and mutant proteins.** Lanes: M, molecular mass marker; 1, the wild-type; 2, the M145A-214A-229A-247L-317I mutant; 3, the M145A-214A-229A-247T-317I mutant; 4, the M145I-214A-229A-247L-317I mutant; 5, the M145I-214A-229A-247T-317I mutant; 6, the M145I-214A-229T-247L-317I mutant; 7, the M145I-214A-229T-247T-317I mutant; 8, the M145A-214A-229T-247L-317I mutant; 9, the M145A-214A-229T-247T-317I mutant.
(DOCX)

Figure S2 **The sequence alignment of the alkaline α-amylase from** ***A. amylolytica*** **(wild-type) and the structure template (AmyB).**
(DOC)

Table S1 **The list of the mutants.**
(DOCX)
