# Supplementary material for: Structure-Guided Systems-Level Engineering of Oxidation-Prone Methionine Residues in Catalytic Domain of an Alkaline α-Amylase from Alkalimonas amylolytica for Significant Improvement of Both Oxidative Stability and Catalytic Efficiency
Source: PLoS One. 2013 Mar 15;8(3):e57403. doi: 10.1371/journal.pone.0057403 (PMC3598850; doi:10.1371/journal.pone.0057403)
Supplement: Figure S1 — The SDS-PAGE analysis of the purified wild-type and mutant proteins. Lanes: M, molecular mass marker; 1, the wild-type; 2, the M145A-214A-229A-247L-317I mutant; 3, the M145A-214A-229A-247T-317I mutant; 4, the M145I-214A-229A-247L-317I mutant; 5, the M145I-214A-229A-247T-317I mutant; 6, the M145I-214A-229T-247L-317I mutant; 7, the M145I-214A-229T-247T-317I mutant; 8, the M145A-214A-229T-247L-317I mutant; 9, the M145A-214A-229T-247T-317I mutant. (DOCX) [file pone.0057403.s001.docx]

**Fig.S1: The SDS-PAGE analysis of the purified wild-type and mutant proteins**

Lanes: M, molecular mass marker; 1, the wild-type; 2, the M145A-214A-229A-247L-317I mutant; 3, the M145A-214A-229A-247T-317I mutant; 4, the M145I-214A-229A-247L-317I mutant; 5, the M145I-214A-229A-247T-317I mutant; 6, the M145I-214A-229T-247L-317I mutant; 7, the M145I-214A-229T-247T-317I mutant; 8, the M145A-214A-229T-247L-317I mutant; 9, the M145A-214A-229T-247T-317I mutant.


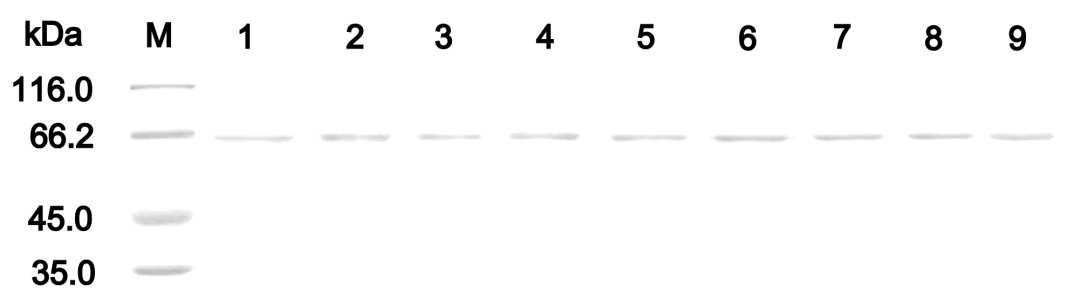


(Fig.S1)
