# Supplementary material for: Structure-Guided Systems-Level Engineering of Oxidation-Prone Methionine Residues in Catalytic Domain of an Alkaline α-Amylase from Alkalimonas amylolytica for Significant Improvement of Both Oxidative Stability and Catalytic Efficiency
Source: PLoS One. 2013 Mar 15;8(3):e57403. doi: 10.1371/journal.pone.0057403 (PMC3598850; doi:10.1371/journal.pone.0057403)
Supplement: Figure S2 — The sequence alignment of the alkaline α-amylase from A. amylolytica (wild-type) and the structure template (AmyB). (DOC) [file pone.0057403.s002.doc]

**Fig.S2. The sequence alignment of the alkaline α-amylase from *A. amylolytica* (wild-type) and the structure template (AmyB)**

The starch binding domain of the alkaline α-amylase from *A. amylolytica* has no identity with that of template α-amylase AmyB (3bc9), and thus the sequence used for alignment is the catalytic domain (Domain A, Domain B and Domain C) of two enzymes. The amino acid sequence (1-96) is the starch binding domain of template α-amylase AmyB (3bc9), and the amino acid sequence (491-588) is the starch binding domain of the alkaline α-amylase from *A. amylolytica*. Since the first 31 amino acids of the alkaline α-amylase from *A. amylolytica* is signal peptide, the mature enzyme was composed of amino acids from 32. The aligned amino acid sequence for the alkaline α-amylase from *A. amylolytica* is from 32 to 491, and that for the template AmyB is from 97 to 587. The same amino acid sequences between the alkaline α-amylase from *A. amylolytica* and the template (AmyB) are highlighted in yellow, and the others are the changed parts.

**

**

(Fig.S2)
