# Supplementary material for: Structure-Guided Systems-Level Engineering of Oxidation-Prone Methionine Residues in Catalytic Domain of an Alkaline α-Amylase from Alkalimonas amylolytica for Significant Improvement of Both Oxidative Stability and Catalytic Efficiency
Source: PLoS One. 2013 Mar 15;8(3):e57403. doi: 10.1371/journal.pone.0057403 (PMC3598850; doi:10.1371/journal.pone.0057403)
Supplement: Table S1 — The list of the mutants. (DOCX) [file pone.0057403.s003.docx]

In this work, 100 mutants were obtained, including 15 one-point mutants and 85 combinational mutants (including 25 double mutants, 36 triple mutants, 16 four-site mutants and 8 five-site mutants). **Table S1: The list of all the mutants**

| One-point mutants | M145T, M214T, M229T, M247T, M317T, M145I, M214I, M229I, M247I, M317I, M145A, M214A, M229A, M247A and M317A |
| --- | --- |
| Double-point mutants | M145I-214A, M145I-229A, M145I-229T, M145I-247T, M145I-247L, M145I-317I, M145A-214A, M145A-229A, M145A-229T, M145A-247T, M145A-247L, M145A-317I, M214A-229A, M214A-229T, M214A-247T, M214A-247L, M214A-317I, M229A-247T, M229A-247L, M229A-317I, M229T-247T, M229T-247L, M229T-317I, M247T-317I and M247L-317I |
| Triple mutants | M145A-214A-229A, M145A-214A-229T, M145A-214A-247T, M145A-214A-247L, M145A-214A-317I, M145I-214A-229A, M145I-214A-229T, M145I-214A-247T, M145I-214A-247L, M145I-214A-317I, M145I-229A-247I, M145I-229A-247T, M145I-229A-317I, M145A-229A-247I, M145A-229A-247T, M145A-229A-317I, M145I-229T-247I, M145I-229T-247T, M145I-229T-317I, M145A-229T-247I, M145A-229T-247T, M145A-229T-317I, M145I-247I-317I, M145I-247T-317I, M145A-247I-317I, M145A-247T-317I, M214A-229A-247T, M214A-229A-247L, M214A-229A-317I, M214A-229T-247T, M214A-229T-247L, M214A-229T-317I, M229T-247T-317I, M229T-247L-317I, M229A-247T-317I and M229A-247L-317I. |
| Four-site mutants | M145A-214A-229A-247L, M145A-214A-229A-247T, M145A-214A-229A-317I, M145I-214A-229A-247L, M145I-214A-229A-247T, M145I-214A-229A-317I, M145A-214A-229T-247L, M145A-214A-229T-247T, M145A-214A-229T-317I, M145I-214A-229T-247L, M145I-214A-229T-247T, M145I-214A-229T-317I, M214A-229A-247L-317I, M214A-229T-247L-317I, M214A-229A-247T-317I and M214A-229T-247T-317I. |
| Five-site mutants | M145A-214A-229A-247L-317I, M145A-214A-229A-247T-317I, M145I-214A-229A-247L-317I, M145I-214A-229A-247L-317I, M145I-214A-229T-247L-317I, M145I-214A-229T-247T-317I, M145A-214A-229T-247L-317I and M145A-214A-229T-247T-317I. |
